# Supplementary material for: Coronary Artery Disease in Patients Older than 35 and Eligible for Cardiovascular Secondary Prevention: An Italian Retrospective Observational Analysis of Healthcare Administrative Databases
Source: J Clin Med. 2021 Oct 14;10(20):4708. doi: 10.3390/jcm10204708 (PMC8540912; doi:10.3390/jcm10204708)
Supplement: Supplementary file 1 [file jcm-10-04708-s001.zip › jcm-1404225-supplementary.pdf]

Figure S1: Percentage distribution of the Italian population in 2015 in the ReS database and according to the Italian Institute of Statistics (ISTAT), by age group

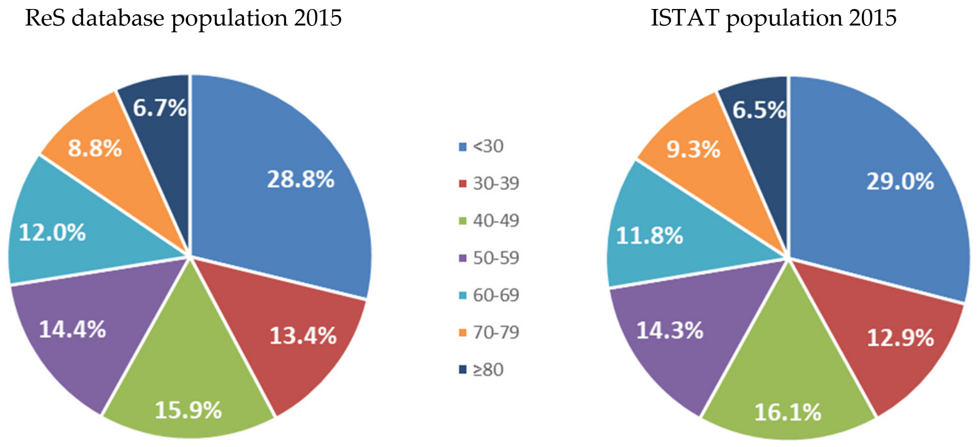

Table S1: Criteria for the selection of patients with coronary artery diseases

Administrative database	Description
**Demographics**	Aged ≥ 35 years
AND at least one of the following criteria	
**Hospitalizations**	Hospitalization with a primary/secondary diagnosis among the following (ICD-9-CM code):<br>410.x – Acute myocardial infarction<br>411.x – Other acute and subacute forms of ischemic heart disease<br>412 – Old myocardial infarction<br>413.x – Angina pectoris<br>414.x - Other forms of chronic ischemic heart disease<br>AND/OR<br>Hospitalization with a procedure on coronary artery among the following (ICD-9-CM code):<br>36.x – Operations on vessels of heart<br>00.66 – PTCA - percutaneous transluminal coronary angioplasty
**Exemption for disease**	002.414 – Diseases of the circulatory system (other forms of chronic ischemic heart disease)<br>A02.414 - Diseases of the circulatory system (other forms of chronic ischemic heart disease)

Table S2: Criteria for the exclusion of patients not eligible for cardiovascular secondary prevention strategies. Patients had to meet at least one of the following criteria per condition in the 3 years before the index date.

**Dialysis therapy**	
**Administrative database**	**Description**
**Hospitalizations**	Hospitalization with a primary/secondary diagnosis among the following (ICD-9-CM code):<br>V56.x - Encounter for dialysis and dialysis catheter care<br>V45.1 - Postsurgical renal dialysis status<br>AND/OR<br>Hospitalization associated to the following DRG (diagnoses related group) code:<br>317 – Hospitalization for renal dialysis<br>AND/OR<br>Hospitalization with a procedure among the following (ICD-9-CM code):<br>39.95 – Hemodialysis<br>54.98 – Peritoneal dialysis
**Outpatient specialist services**	39.95 – Hemodialysis<br>54.98 – Peritoneal dialysis
**Neoplasia**	
**Administrative database**	**Description**
**Hospitalizations**	Hospitalization with a primary/secondary diagnosis among the following (ICD-9-CM code):<br>From 140.x to 208.x - Neoplasms<br>V10.x - Personal history of malignant neoplasm<br>V58.1x – Chemotherapy<br>AND/OR<br>Hospitalization with a procedure among the following (ICD-9-CM code):<br>00.10 – Implantation of chemotherapeutic agent<br>99.25 – Injection or infusion of cancer chemotherapeutic substance<br>99.28 - Injection or infusion of biological response modifier [BRM] as an antineoplastic agent
**Outpatient specialist services**	99.25 - Injection or infusion of cancer chemotherapeutic substance
**Exemption for disease**	048 – Patients affected by malignant neoplasms and by tumors of uncertain behavior
**Pharmaceuticals**	A prescription of a specific drug:<br>L01 – Antineoplastic agents<br>AND/OR<br>A prescription with the exemption code 048

Table S3: Criteria for the selection of patients with relevant comorbidities during the available period before the index date. Patients had to meet at least one of the following criteria per comorbidity.

Heart failure	
**Administrative database**	**Description**
**Hospitalizations**	Hospitalization with one of the following main/secondary diagnoses (ICD-9-CM code):<br>402.01 – Malignant hypertensive heart disease with heart failure<br>402.11 – Benign hypertensive heart disease with heart failure<br>402.91 – Unspecified hypertensive heart disease with heart failure<br>404.01 – Hypertensive heart and chronic kidney disease, malignant, with heart failure and with chronic kidney disease stage I through stage IV, or unspecified<br>404.03 – Hypertensive heart and chronic kidney disease, malignant, with heart failure and with chronic kidney disease stage V or end stage renal disease<br>404.11 – Hypertensive heart and chronic kidney disease, benign, with heart failure and with chronic kidney disease stage I through stage IV, or unspecified<br>404.13 – Hypertensive heart and chronic kidney disease, benign, with heart failure and chronic kidney disease stage V or end stage renal disease<br>404.91 – Hypertensive heart and chronic kidney disease, unspecified, with heart failure and with chronic kidney disease stage I through stage IV, or unspecified<br>404.93 - Hypertensive heart and chronic kidney disease, unspecified, with heart failure and chronic kidney disease stage V or end stage renal disease<br>428.x – Heart failure
**Exemption for disease**	021.428 - Heart failure
**Pharmaceuticals**	A prescription of the following specific drug:<br>C09DX04 – Sacubitril/Valsartan<br>AND/OR<br>A prescription with the exemption for disease code 021
**Atrial fibrillation**	
**Administrative database**	**Description**
**Hospitalizations**	Hospitalization with the following main/secondary diagnosis (ICD-9-CM code):<br>427.3x - Atrial fibrillation and flutter
**Cerebrovascular diseases**	
**Administrative database**	**Description**
**Hospitalizations**	Hospitalization with one of the following main/secondary diagnoses (ICD-9-CM code):

	430 – 438.x - Cerebrovascular Disease
**Depression**	
**Administrative database**	**Description**
**Hospitalizations**	Hospitalization with one of the following primary/secondary diagnoses (ICD-9-CM code):<br>296.2x - Major depressive disorder single episode<br>296.3x - Major depressive disorder recurrent episode<br>296.5x - Bipolar disorder, most recent episode (or current) depressed<br>296.82 - Atypical depressive disorder<br>298.0x - Depressive type psychosis<br>300.4 - Dysthymic disorder<br>301.12 - Chronic depressive personality disorder
**Exemption for disease**	044.296.2 - Psychosis (major depressive disorder single episode)<br>044.296.3 - Psychosis (major depressive disorder recurrent episode)<br>044.296.5 - Psychosis (bipolar disorder, most recent episode (or current) depressed)<br>044.296.8 - Psychosis (manic depressive psychosis, other unspecified)<br>044.298.0 – Psychosis (depressive type psychosis)
**Pharmaceuticals**	A prescription of a specific drug (ATC code):<br>N06A– Antidepressants
**Diabetes**	
**Administrative database**	**Description**
**Hospitalizations**	Hospitalization with the following primary/secondary diagnosis (ICD-9-CM code):<br>250.x - Diabetes mellitus
**Exemption for disease**	013.250 - Diabetes mellitus
**Pharmaceuticals**	A prescription of a specific drug (ATC code):<br>A10 - Drugs used in diabetes<br>AND/OR<br>A drug prescription with the exemption for disease code 013
**Dyslipidaemia**	
**Administrative database**	**Description**
**Hospitalizations**	Hospitalization with the following primary/secondary diagnosis (ICD-9-CM code):<br>272.x - Disorders of lipid metabolism
**Exemption for disease**	025 - Type IIa and IIb heterozygous familial hypercholesterolemia – Polygenic hypercholesterolemia – Familial combined hypercholesterolemia – Type III hyperlipoproteinemia

**Pharmaceuticals**	Prescription of at least 3 packs of specific drugs:<br>C10A - Lipid modifying agents, plain<br>C10B - Lipid modifying agents, combinations<br>AND/OR<br>A prescription with the exemption for disease code 025
**Arterial hypertension**	
**Administrative database**	**Description**
**Hospitalizations**	Hospitalization with one of the following primary/secondary diagnoses (ICD-9-CM code):<br>401.x - Essential hypertension<br>402.x - Hypertensive heart disease<br>403.x - Hypertensive chronic kidney disease<br>404.x - Hypertensive heart and chronic kidney disease<br>405.x - Secondary hypertension
**Exemption for disease**	031 – Arterial hypertension<br>A31 - Arterial hypertension without organ damage
**Pharmaceuticals**	Prescription of <u>at least 4 packs</u> of one or more specific drugs (ATC code):<br>C02 – Antihypertensives<br>C03 - Diuretics<br>C07 – Beta blocking agents<br>C08 – Calcium channel blockers<br>C09 – Agents acting on the renin-angiotensin system<br>AND/OR<br>A prescription with the exemption for disease code 031 or A31
**Severe liver diseases**	
**Administrative database**	**Description**
**Hospitalizations**	Hospitalization with one of the following main/secondary diagnoses (ICD-9-CM code):<br>070.x - Viral hepatitis<br>571.x - Chronic liver disease and cirrhosis<br>572.x - Liver abscess and sequelae of chronic liver disease<br>573.x - Other disorders of liver<br>V42.7 - Liver replaced by transplant
**Exemption for disease**	016 – Chronic hepatitis (active)<br>008 – Liver cirrhosis, biliary cirrhosis
**Pharmaceutical**	A prescription of a specific drug (ATC code):<br>J05AP - Antivirals for treatment of HCV infections<br>J05AF08 - adefovir<br>J05AF10 - entecavir<br>J05AF11 - telbivudine<br>AND/OR<br>A prescription with the exemption for disease code 016 or 008

Chronic lung diseases	
**Administrative database**	**Description**
**Hospitalizations**	Hospitalization with one of the following main/secondary diagnoses (ICD-9-CM code):<br>490.x - Bronchitis, not specified as acute or chronic<br>491.x - Chronic bronchitis<br>492.x - Emphysema<br>493.x - Asthma<br>494.x - Bronchiectasis<br>496.x - Chronic airway obstruction, not elsewhere classified<br>518.81- 518.84 - Respiratory failure
**Exemption for disease**	024 – Chronic respiratory failure<br>007 - Asthma
**Pharmaceutical**	Prescription of at least 3 packs of drugs for obstructive airway diseases:<br>R03 – Drugs for obstructive airway diseases<br>AND/OR<br>Prescription with the exemption for disease code 024 or 007
**Rheumatoid arthritis**	
**Administrative database**	**Description**
**Hospitalizations**	Hospitalization with a primary/secondary diagnosis among the following (ICD-9-CM code):<br>714.0 – Rheumatoid arthritis<br>714.1 – Felty's syndrome<br>714.2 – Other rheumatoid arthritis with visceral or systemic involvement<br>714.3x - Juvenile chronic polyarthritis
**Exemption for disease**	006 - Rheumatoid arthritis

Table S4: Criteria for the identification of cardiovascular relevant causes of hospitalization in the follow-up.

Acute coronary syndrome	
**Administrative database**	**Description**
**Hospitalizations**	Hospitalization with a primary/secondary diagnosis among the following (ICD-9-CM code):<br>410.x - Acute myocardial infarction<br>411.x – Other acute and subacute forms of ischemic heart disease
**Angina pectoris**	
**Administrative database**	**Description**
**Hospitalizations**	Hospitalization with the following primary/secondary diagnosis the following (ICD-9-CM code):<br>413.x – Angina pectoris
**Heart failure**	
**Administrative database**	**Description**
**Hospitalizations**	Hospitalization with one of the following main/secondary diagnoses (ICD-9-CM code):<br>402.01 – Malignant hypertensive heart disease with heart failure<br>402.11 – Benign hypertensive heart disease with heart failure<br>402.91 – Unspecified hypertensive heart disease with heart failure<br>404.01 – Hypertensive heart and chronic kidney disease, malignant, with heart failure and with chronic kidney disease stage I through stage IV, or unspecified<br>404.03 – Hypertensive heart and chronic kidney disease, malignant, with heart failure and with chronic kidney disease stage V or end stage renal disease<br>404.11 – Hypertensive heart and chronic kidney disease, benign, with heart failure and with chronic kidney disease stage I through stage IV, or unspecified<br>404.13 – Hypertensive heart and chronic kidney disease, benign, with heart failure and chronic kidney disease stage V or end stage renal disease<br>404.91 – Hypertensive heart and chronic kidney disease, unspecified, with heart failure and with chronic kidney disease stage I through stage IV, or unspecified<br>404.93 - Hypertensive heart and chronic kidney disease, unspecified, with heart failure and chronic kidney disease stage V or end stage renal disease<br>428.x – Heart failure
**Haemorrhagic stroke/ intracranial haemorrhage**	
**Administrative database**	**Description**
**Hospitalizations**	Hospitalization with one of the following main/secondary diagnoses (ICD-9-CM code):<br>430.x – Subarachnoid haemorrhage<br>431.x – Intracerebral haemorrhage<br>432.x – Other and unspecified intracranial haemorrhage
**Ischemic stroke/transient ischemic attack**	

Administrative database	Description
**Hospitalizations**	Hospitalization with one of the following main/secondary diagnoses (ICD-9-CM code):<br>433.x – Occlusion and stenosis of precerebral arteries<br>434.x – Occlusion of cerebral arteries<br>435.x – Transient cerebral ischemia<br>436 – Acute but ill-defined cerebrovascular disease<br>437.x - Other and ill-defined cerebrovascular disease

Table S5: Hospitalizations of patients with CAD and eligible for cardiovascular (CV) secondary prevention drugs during the follow-up year

	Patients hospitalized (n; %)	Mean number of hospitalizations per hospitalized patient	Mean number of days of in-hospital stay
Main diagnoses identifying the relevant CV events			
Acute coronary syndrome (ACS)	2,392; 5.2	1.2	8.2
Heart failure	1,847; 4.0	1.4	10.7
Angina pectoris	741; 1.6	1.0	4.5
Ischemic stroke/transient ischemic attack (TIA)	652; 1.4	1.1	9.0
Haemorrhagic stroke/intracranial bleeding	61; 0.1	1.1	10.8
At least a hospitalization due to relevant CV events	5,244; 11.4	1.3	8.8
Main diagnoses identifying other CV events (first 5 in descending order)			
Chronic ischemic heart disease, other than old myocardial infarction and angina pectoris	2,174; 4.7	1.1	7.8
Ill-defined descriptions and complications of heart disease	875; 1.9	1.0	16.9
Cardiac dysrhythmias	576; 1.2	1.2	6.1
Atherosclerosis	369; 0.8	1.3	9.0
Other diseases of endocardium	356; 0.8	1.2	12.9
At least a hospitalization due to other CV events	4,185; 9.1	1.2	9.7
Main diagnoses identifying non-CV causes of hospitalization (first 5 in descending order)			
Diseases of lung, other than acute pulmonary infections, lung diseases due to external agents and chronic obstructive pulmonary diseases	1,019; 2.2	1.3	12.0
Organ or tissue replaced by artificial/mechanical devices/prosthesis	493; 1.1	1.0	19.9
Symptoms involving respiratory system and other chest symptoms	473; 1.0	1.0	4.5
Acute renal failure	318; 0.7	1.1	10.9
Cholelithiasis	240; 0.5	1.2	9.6
At least a hospitalization due to non-CV causes of hospitalization	8,053; 17.5	1.5	11.1
